# From policy to practice and vice versa: a cross-sectional study of healthcare workers’ perspectives on safeguarding public health service access for displaced populations in Iran

**DOI:** 10.3389/fpubh.2026.1737982

**Published:** 2026-02-18

**Authors:** Zahra Karimian, Asgar Aghaei Hashjin, Saverio Bellizzi, Volker Winkler

**Affiliations:** 1 Heidelberg Institute of Global Health (HIGH), Faculty of Medicine and University Hospital, Heidelberg University, Heidelberg, Germany; 2 School of Health Management and Information Sciences, Iran University of Medical Sciences (IUMS), Tehran, Iran; 3 Public Health and Migration, World Health Organization Headquarters (WHO HQ), Geneva, Switzerland

**Keywords:** displaced populations, Eastern Mediterranean Region, equitable access to health care, healthcare workers, migrants and refugees, provider perspectives, public health services, universal health coverage

## Abstract

**Background:**

Global progress toward the Sustainable Development Goals (SDGs) is severely off track. Iran hosts the world’s largest migrant and refugee population, but sanctions, inflation, and workforce migration have strained its capacity. Since the attitudes of service providers influence whether inclusive policies are translated into practice, this study explores frontline staff perspectives on access to public health services for non-citizens in one of Tehran’s largest public health networks.

**Methods:**

We conducted a cross-sectional survey among staff in three public health centers that serve migrant and refugee populations. The questionnaire assessed five Likert scale outcomes: support for separate centers, shared user fees, prioritization of citizens under conditions of scarcity, equal service provision, and preference for working with citizens. Demographic variables included age, sex, years of experience, and role (clinical vs. non-clinical). Items were analyzed descriptively; subgroup comparisons were conducted using two-sided Mann–Whitney U tests.

**Results:**

Of approximately 150 eligible healthcare workers, 87 provided complete responses. Agreement with prioritizing citizens was highest at 93.1% (median 5). Support for equal service provision was lowest, with 56.3% of participants expressing disagreement (median 2). A preference for working with citizens was endorsed by 61.9% of the respondents (median 4) and differed by age, experience, and role, with younger, less-experienced, and clinical staff reporting stronger preferences (all *p* < 0.01).

**Conclusion:**

Healthcare workers’ perspectives can serve as a rapid, low-burden proxy indicator for equitable service delivery. In this exploratory study, workers favored citizen prioritization and fee-based models under conditions of scarcity, with the lowest support expressed for equal access for non-citizens; these preferences were strongest among younger and clinical staff. Adapted service delivery models, targeted training, and clear operational guidance are needed to support equitable access in resource-constrained settings.

## 1 Background

In 2024, global progress toward the Sustainable Development Goals (SDGs) was reported to be “severely off track,” with targets linked to health (SDG 3), education (SDG 4), and reducing inequalities (SDG 10) found to be stagnating or even regressing (1). At the same time, ongoing conflicts, political and economic crises, and environmental changes continue to drive unprecedented levels of forced displacement, further impeding progress toward SDGs (2). Recent reports from the United Nations High Commissioner for Refugees (UNHCR) estimate that more than 123 million people were forcibly displaced worldwide in 2024 (3), with the highest concentrations in the Eastern Mediterranean Region (EMR) (4). This placed additional pressure on the health and education infrastructures of host countries in the region (5).

Among EMR member states, Iran hosts the world’s largest population of migrants and refugees, predominantly from Afghanistan (6). However, with heightened regional tensions and security concerns, Iranian authorities have sharply increased deportations of Afghan migrants and refugees since 2024, with numbers rising further in 2025 (7). At the same time, international agencies have warned that forced returns place severe strain on Afghanistan’s already fragile systems and pose urgent humanitarian and public health risks for returnees (8–10).

Despite these challenges, Iran’s healthcare system has remained comparatively inclusive, granting access to migrants, refugees, and even undocumented individuals (UIs) (11). The country’s public health system is organized around a nationwide primary healthcare network that integrates community-based health facilities in rural areas, public health centers in urban areas, and referral hospitals in major cities (12). These facilities provide a wide range of preventive, promotive, and curative services, including maternal and child healthcare, vaccination programs, communicable and non-communicable disease management, rehabilitation services, and health promotion campaigns (13, 14). Clinical services at public health centers are complemented by school-based health activities conducted during visits to public schools. These activities include health education, vision and hearing screenings, dental hygiene checks, nutrition assessments, head-lice checks, and referrals to social services for children who may be affected by violence or abuse (15). Notably, both public health and education services are available to Afghan migrants and refugees (16–18). However, sanctions, economic pressures, and the out-migration of health professionals have stretched the system’s capacity to sustain inclusive access (19). From a population health standpoint, these pressures have direct implications for national policy implementation aimed at achieving universal health coverage (UHC) and contribute to setbacks in meeting international commitments to SDG targets (20, 21).

Ensuring health access for all also depends on the perspectives and attitudes of frontline healthcare workers, since provider willingness to engage with patients, particularly non-citizens, influences whether inclusive public health policies can be translated into equitable service delivery in practice (22). Therefore, staff perspectives constitute a modifiable determinant of population health and serve as a potential indicator for evaluating and monitoring equitable service delivery over time (23). This approach aligns with the World Health Organization (WHO) recommendation to establish national health inequality monitoring systems that identify disadvantaged subgroups, track and analyze disaggregated data, and use evidence to inform policies and programs, ensuring that no one is left behind (24).

In this context, we used healthcare worker perspectives as a proxy indicator of equitable service delivery, contributing a data point that can complement the broader monitoring framework envisaged in the WHO’s step-by-step manual (24). Conceptually, we used a policy-to-practice translation framework in which national policy and resource constraints influence facility operations and implementation conditions, while provider attitudes shape point-of-care decisions and behaviors, with downstream effects on service delivery experiences, utilization, and equity outcomes (25).

We previously analyzed patient records from the largest public health network in the capital city and observed a decline in the proportion of Afghan patients receiving full health coverage following the 2021 crisis in Afghanistan and the subsequent influx of migrants to Iran (26). This retrospective study highlighted the patient subgroups experienced the greatest decline in utilization. The present cross-sectional study focuses on healthcare workers within the same network to explore whether provider perspectives may represent a plausible contributor to the previously observed decline in utilization among Afghan patients, alongside broader structural and operational pressures.

For this purpose, we designed a brief survey to explore healthcare workers’ perspectives on five key policy-relevant domains—separation of service centers, shared user fees, prioritization when resources are scarce, equal provision of healthcare, and staff preferences regarding patient groups—and to assess whether responses vary by demographic characteristics (sex and age) and professional characteristics (work experience and professional role). By assessing provider perspectives, we aimed to identify modifiable barriers, inform targeted capacity-building initiatives, and prioritize the allocation of supportive measures (27, 28).

## 2 Methods

### 2.1 Study design and setting

We conducted a brief, cross-sectional survey in September 2024 at three public health centers within one of the largest public health networks in the capital city of Iran, which serves approximately 5.5 million residents in western Tehran, including nearly one million Afghans (29). The participating centers provide primary healthcare to catchment areas with substantial migrant and refugee populations.

### 2.2 Participants and recruitment

Convenience sampling was employed to include as many participants as possible in the survey. All on-site healthcare staff members at the three public health centers were invited to participate during the data collection period (approximately 150 eligible staff). The inclusion criteria included employment at one of the centers, regardless of direct or indirect involvement in patient care. Staff members with direct patient contact were classified as clinical; staff members supporting service delivery without direct patient contact were classified as non-clinical. Clinical roles included physicians, midwives, nurses, dentists, pharmacists, nutritionists and dietitians, social workers, and psychologists. Non-clinical roles included community health workers, public health practitioners, health educators, and facilitators, as well as administrative and management staff supporting organizational and financial operations. Clinical staff at public health centers typically work six 7-h shifts per week, while non-clinical staff work five 8-h shifts per week, either on-site at the public health centers or in off-site offices. The exclusion criteria included non-response or incomplete surveys.

### 2.3 Questionnaire development

A brief questionnaire was developed to assess frontline workers’ perspectives that may affect service delivery. Instrument brevity was prioritized to minimize respondent burden and maximize completion in busy, resource-constrained clinics; longer questionnaires can increase response burden and missing data in applied settings (30, 31). The questionnaire included five policy-relevant items measured on a 5-point Likert scale (1 = completely disagree, 5 = completely agree), covering the following domains: Dedicating separate service centers for migrants and refugees (Q1), charging separate fees for public health services offered to migrants and refugees (Q2), prioritizing citizens (taxpayers) when under resource constraints (Q3), providing equal services to citizens and non-citizens (Q4), and personal preference for working with citizens (Q5). The items were intentionally mixed in direction, with Q4 phrased opposite to the other statements, to enable attention screening. The domains were selected *a priori* based on evidence from Iran, other low- and middle-income countries (LMICs), and international policy reviews (32–34) and were refined with input from facility managers during instrument development. This source triangulation strengthened content validity. Furthermore, three management-level staff also reviewed the questionnaire for face validity and cultural appropriateness, after which the research team made minor wording refinements to ensure clarity before administering the survey in Persian. Table 1 presents English back-translations of the survey items and their intended objectives.

**Table 1:** Objectives and survey items formulated as policy statements.

Objectives	Survey items/questions (Q1–5)
This item probes whether staff favor segregating/separating services by population group.	Q1. “Separate centers/facilities should be established for providing health services to citizens versus migrants and refugees.”
This gauges support for requiring payment from migrants and refugees, who could otherwise access subsidized public health services at the same cost as citizens without health insurance.	Q2. “A separate fee should be charged for public health services provided to migrants and refugees compared to citizens.”
This evaluates attitudes toward prioritizing public services for taxpaying citizens under resource constraints.	Q3. “In times of limited resources, priority in public services should be given to citizens (taxpayers) over migrants/refugees.”
This assesses support for equity and non-discrimination in service provision regardless of citizenship or legal residence status.	Q4. “The same public health services and equal access should be provided to both citizens and migrants/refugees.”
This captures personal preference for working with individuals from a different language, cultural, and/or religious background (foreign nationals).	Q5. “I prefer working with citizens (as clients/patients) rather than with migrants/refugees.”

English back-translations of survey items and their objectives, framed as policy statements.

Respondents indicated agreement on a 5-point Likert scale (1 = “Completely Disagree,” 2 = “Partially Disagree,” 3 = “Neither Agree nor Disagree,” 4 = “Partially Agree,” and 5 = “Completely Agree”).

### 2.4 Data collection (variables and quality checks)

Collected demographic variables included age, sex, years of work experience, and professional role (clinical vs. non-clinical). The primary outcomes were the five Likert-item responses. We planned a complete case analysis for the five items. Prior to analysis, we screened for inattentive responding (“straight-lining”) by flagging uniform response patterns across items, given that the items were designed with mixed directionality (35).

### 2.5 Statistical analysis

We treated Likert responses as ordinal. For each item, we described the full distribution (counts and percentages for categories 1–5) and reported the median (IQR) and mean (with means presented for descriptive purposes only). Subgroup comparisons were conducted using two-sided Mann–Whitney U tests for each item across the four demographic and occupational variables (age, sex, years of work experience, and professional role). We dichotomized age and years of experience at the sample medians to enable two-group nonparametric tests and avoid multiple categories with low counts. This approach was chosen to simplify presentation for policymakers and decision-makers (36). Professional role was coded as clinical versus non-clinical, and sex as female versus male. Statistical significance was evaluated at a two-sided *p*-value of <0.05. To address multiple testing, a Bonferroni correction was planned as a sensitivity analysis. Analyses were conducted using the R software (version 4.4.1).

### 2.6 Ethics and reporting

The study protocol followed institutional ethical requirements for survey research. Participation was voluntary and anonymous. Informed consent was implied through participation, as choosing to decline had no consequences. We report the results in accordance with the STROBE guidelines for cross-sectional studies (37).

## 3 Results

Respondents completed the survey anonymously on paper, and the data were entered into Excel, cleaned, and restructured before being analyzed in R. Four surveys were excluded: one due to missing demographic information and three for uniform responses across all five items (“straight-lining”). After exclusion, 87 out of approximately 150 eligible staff provided complete surveys (58% response). The mean age of participants was 39.7 years (SD 9.9), 85.1% were women, 60.9% were clinical staff, and the median work experience was 15 years [IQR 5–21]. The participant characteristics are summarized in Table 2.

**Table 2:** Participant characteristics (*N* = 87).

Characteristic	Summary
Age (years)	Mean 39.7 (SD 9.9)
	Median 39 [IQR 30.0–46.5]
	Range 23–65
Sex	Female: 74 (85.1%)
	Male: 13 (14.9%)
Work experience (years)	Mean 14.6 (SD 10.1)
	Median 15.0 [IQR 5.0–21.0]
	Range 0–40
Professional role	Clinical staff: 53 (60.9%)
	Non-clinical staff: 34 (39.1%)

Demographic and professional characteristics of the survey sample (*N* = 87).

**Figure 1:**
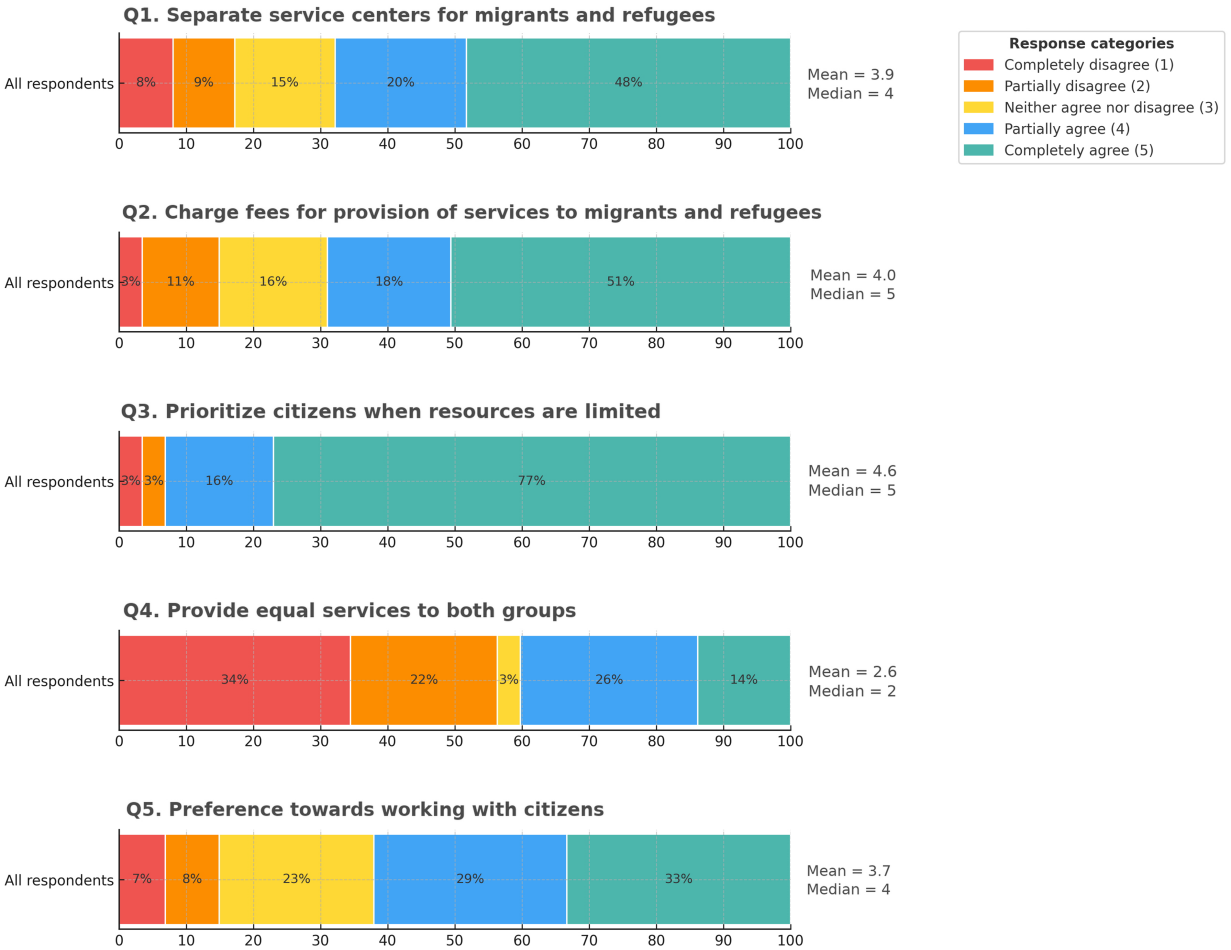
Distribution of healthcare worker attitudes across the five policy domains (*N* = 87). Stacked bars show response percentages for Likert categories 1–5; means and medians are marked. Higher scores reflect less inclusive views for Q1, Q2, Q3, and Q5 and a more inclusive position for Q4.

Across all respondents (*N* = 87), Q1 (separate service centers) showed partial agreement (mean 3.91, median 4), indicating moderate support with some dissent. Q2 (fee-based measures for public services offered to migrants and refugees) showed stronger support (mean 4.01, median 5). Q3 (prioritizing citizens when resources are limited) received the strongest endorsement (mean 4.60, median 5) with no neutral responses, suggesting a clear stance on prioritization under conditions of scarcity. In contrast, Q4 (providing equal services to both groups) showed the lowest support or highest disagreement (mean 2.63, median 2). Q5 (preference for working with citizens) showed heterogeneous views (mean 3.74, median 4). The response distributions (1–5), along with means and medians, are depicted in Figure 1.

Subgroup analyses using two-sided Mann–Whitney U tests are presented in Table 3. For Q1–Q4, no subgroup comparisons reached significance (all *p* > 0.05). Only Q5 differed between the subgroups. Higher scores indicate a preference for working with citizens. Scores were higher among younger staff (≤39 vs. >39 years; medians 5 vs. 3; *p* < 0.001) and among those with less work experience (≤15 vs. >15 years; medians 5 vs. 3; *p* < 0.001), consistent with the close correlation between age and years of work experience. Based on professional role, clinical staff scored higher than non-clinical staff (medians 4 vs. 3; *p* = 0.0024). No differences were observed by sex (median 4 for both male and female respondents; *p* = 0.80). After applying the Bonferroni threshold as a sensitivity check, the set of statistically significant results remained unchanged.

**Table 3:** Mann–Whitney U test results (two-sided) for subgroup differences in healthcare worker perspectives by sex, age, years of work experience, and professional role.

Survey item	Sex (female vs. male; *n* = 74, 13)	Age (≤39 vs. >39; *n* = 48, 39)	Work experience (≤15 vs. >15; *n* = 48, 39)	Professional role (clinical vs. non-clinical; *n* = 53, 34)
Q1. Separate service centers for migrants and refugees	0.18	0.83	0.76	0.57
Q2. Separate fee for public health services to non-citizens	0.46	0.16	0.21	0.37
Q3. Prioritize citizens when resources are limited	0.43	0.87	0.53	0.25
Q4. Provide or offer same/equal services to both groups	0.31	0.15	0.26	0.83
Q5. Preference for working with citizens	0.80	<0.001*	<0.001*	0.0024*

Two-sided Mann–Whitney U test results indicating whether differences in healthcare worker perspectives were statistically significant across subgroups defined by sex, age, years of work experience, and professional role.

Age and work experience were dichotomized at the sample medians (age ≤39 vs. >39 years; experience ≤15 vs. >15 years). Professional role was coded as clinical versus non-clinical, and sex as female versus male. *p*-values are from two-sided Mann–Whitney U tests. Results significant at *p* < 0.05 are shown in bold. Asterisks (*) indicate results that remained significant after Bonferroni correction for multiple comparisons.

## 4 Discussion

### 4.1 Key findings and interpretation

#### 4.1.1 Overall patterns

In this survey of healthcare workers’ perspectives on the provision of public health services to non-citizens in Iran, we noted a prevalent view that non-citizens should receive some differentiated access to public health services compared to citizens. The vast majority of respondents supported prioritizing citizens when resources are scarce, and approximately half endorsed separate service delivery infrastructure and fee-based approaches for migrants and refugees. Support for providing equal services to both groups was the lowest across the five domains. These findings highlight a policy–practice gap between frontline perspectives and the inclusive ideals promoted in international frameworks such as the SDGs and UHC. While Iran’s policy stance has been inclusive for decades (for example, access for refugees and UIs to public health and education) (38), respondents often perceived full inclusion to be difficult to achieve and sustain under current constraints. This pattern is consistent with reports from Jordan and Lebanon, where healthcare providers have also expressed resource-driven concerns in caring for Syrian and Palestinian refugees (39, 40). The inclination to prioritize citizens under conditions of scarcity can be understood in terms of fairness and social contract considerations, as many healthcare providers believe that limited public resources should first secure care for their own communities (41, 42). Similar patterns have been described in other host settings, where humanitarian commitments coexist with concerns about local access and system capacity (43). Perspectives may also reflect daily operational pressures identified in previous studies, such as facility crowding, heavier workloads, longer wait times, and communication barriers (23). These pressures may help explain why some staff favored policies perceived to relieve strain, such as separate service centers for migrant and refugee patients or fee-based mechanisms to offset costs. Taken together with prior analysis showing reduced utilization among Afghan migrant and refugee patients (26), the present findings suggest that provider perceptions and behaviors under conditions of scarcity may contribute to declines in service utilization, alongside broader structural constraints.

#### 4.1.2 Subgroup comparisons

Age, work experience, and professional role were associated with differences in attitude intensity. Although age and years of experience are highly correlated, they are conceptually distinct, and separating the effects of age from work experience on performance and competency-based outcomes in clinical practice can be complex (44). For example, recent graduates may be more familiar with digital tools and more up to date with guidelines despite having less work experience (45, 46). Our findings showed that younger staff and those with fewer years of experience were more likely to express a preference for working with citizens than older, more experienced colleagues and that clinical staff expressed stronger preferences than non-clinical staff. Possible explanations for these generational differences include limited experience with migrant patients and the challenging economic and sociopolitical contexts in which younger professionals have trained (47). The differences observed based on professional role merit qualitative exploration to better understand the experiences that shape staff perspectives and inform targeted interventions, including migrant- and refugee-focused staff training, on-demand access to interpreters, and the establishment of volunteer programs to support new arrivals in navigating the host health system without increasing clinical workload (48). Given the small number of male individuals in the sample, sex subgroup comparisons should be interpreted cautiously (as exploratory only).

### 4.2 Policy and practice considerations

#### 4.2.1 Adapted service delivery models

Currently, migrants and refugees have access to the same public health centers as citizens and receive the same type and level of care (32). Separate centers for migrants and refugees could institutionalize segregation if implemented as parallel systems. However, time-limited pilots of specialized clinic sessions within existing facilities, staffed by trained teams and governed by clear referral criteria, may improve efficiency and support quality without creating a second-tier system (49, 50). Alternatively, establishing co-located “one-stop” hubs within existing public health facilities could provide additional support services, such as interpreters, patient navigators, and legal and social assistance for foreign nationals (51). Furthermore, integrated models can be strengthened by offering additional supportive measures during peak hours, including appointment systems with fast-track lanes for immunization and antenatal care in clinics with high refugee caseloads (52). These measures may relieve pressures without the need for physical separation.

#### 4.2.2 Increased investment and resource allocation

Strengthening system capacity through dedicated funding streams for high-volume sites, surge staffing, coordinated volunteer programs, and mobile outreach in areas with many newcomers can help ensure that serving refugees is not perceived as coming at the expense of public healthcare for tax-paying citizens (53). In addition, workable models, such as tailored insurance schemes for asylum seekers and UIs, can further reduce financial barriers to accessing public healthcare (54). When healthcare staff see that the system can accommodate all patients, they are more likely to support inclusive policies. To enable these investments, global guidance recommends pairing humanitarian financing with domestic health system investment to protect equity during refugee crises (55). Importantly, international health agencies and non-profits play a crucial role in co-financing and scaling up capacity in regions with high numbers of migrants and refugees, as well as in procuring supplies and tools (for example, bilingual materials, interpreter services, and emergency medicine kits), which can help ease pressure on frontline workers (56).

#### 4.2.3 Appropriate training programs

Incorporating training modules on migrant health and culturally sensitive and appropriate care into medical and nursing curricula and continuing education programs for licensed staff could better prepare providers and help shift perspectives toward this patient population, particularly among younger professionals (57–59). Migrant and refugee populations have unique needs and face distinct barriers such as lower health literacy, exposure to violence and conflict, disruptions in continuity of care, legal uncertainty, and financial constraints—all of which can make their health assessment, communication, and follow-up complex—warranting specialized training for all healthcare workers (60, 61). Supportive measures such as readily available interpreters and care coordinators to help patients navigate the host health system are not substitutes for this training; rather, they complement it (62).

#### 4.2.4 Operational guidance

Brief surveys of frontline healthcare workers can provide timely, low-burden input to inform feasible short-term operational recommendations in resource-constrained settings where rapid information is needed (63, 64). Based on our findings, we suggest practical steps for facility managers that could be implemented within 6–12 months, including standardized facility guidance, brief refresher training, and supportive supervision to strengthen consistent policy implementation. Clear communication of the public health rationale for inclusive primary care, and its benefits for the wider population and the healthcare system, may further support uptake of these recommendations and improve implementation consistency among frontline workers (65, 66).

### 4.3 Strengths and limitations

This exploratory cross-sectional survey used a brief, targeted instrument (questionnaire) that was feasible in busy clinics, improving participation and attention to items. However, brevity limited scope and nuance. Although staff perspectives serve as an informative proxy, they cannot replace direct measures of health inequities among patient population subgroups. Moreover, although single-item Likert measures enabled rapid assessment, they do not capture the full complexity of attitudes, underscoring the need for a subsequent qualitative phase (67). Additionally, we did not conduct a test–retest reliability assessment due to time constraints during instrument development. Although the sample included staff from three urban public health centers serving high refugee caseloads, with a relatively high response rate, representativeness is limited, and the findings are likely not generalizable, particularly to rural or private-sector settings. We note that subgroup comparisons were simplified by dichotomizing age and years of experience at the medians to maintain adequate group sizes and support policy-relevant interpretation for management, acknowledging that this reduces information relative to continuous analyses. Finally, since this is a cross-sectional snapshot taken during a specific period of regional instability, these pressures and related policies may change over time, and provider perspectives may shift accordingly (68). Beyond these methodological limitations, our findings should be interpreted in light of broader contextual factors that may confound the relationship between provider perspectives and service use. Sanctions, inflation, and economic instability can constrain budgets, staffing, and medicine availability, increasing the workload and potentially influencing provider attitudes toward inclusive service provision. Increased enforcement and deportation pressures may additionally deter care-seeking behavior and contribute to reduced service use among refugees and undocumented individuals (69, 70).

### 4.4 Future research

The next research phase of this sequential explanatory mixed-methods approach will include focus group discussions and/or semi-structured in-depth interviews with healthcare workers as well as patients to explain observed patterns using purposive sampling to capture key perspectives (71). Core domains will include perceived fairness and ethical tensions during scarcity, workload and crowding, communication barriers, clarity of facility guidance, experiences with migrant/refugee patients, and recommendations for service-delivery adaptation. Qualitative data will be analyzed using reflexive thematic analysis to identify and interpret recurrent themes related to attitudes and implementation challenges (72). Together, the survey and interviews will constitute a more holistic approach, strengthening interpretation and the practical utility of the results.

## 5 Conclusion

Health workers play a pivotal role in fulfilling international commitments to UHC for displaced populations. In this cohort of healthcare workers, we observed a strong inclination to prioritize citizens when resources are scarce, with mixed views on structural interventions, and the lowest support for equal provision of public health services to both citizens and non-citizens. Preferences were most pronounced among younger and clinical staff. Provider perspectives and attitudes toward access to care during periods of scarcity can serve as a useful proxy indicator of whether inclusive national policies can be translated into equitable service delivery. Addressing these perspectives through adapted service delivery models, targeted training programs, increased resource allocation for supportive measures, and clear operational guidance on access to public health services for displaced populations, particularly during periods of scarcity, could help align practice with policy goals while acknowledging local constraints. The insights from this survey can inform the next phase of qualitative research and policy strategies aimed at ensuring that “*no one is left behind,*” even in contexts where resources are increasingly limited (73).

## Data Availability

The data in this exploratory study are part of a survey which was administered to healthcare workers at the IUMS Public Health Network. The dataset compiled from their responses is not publicly available but may be made available from the corresponding author on reasonable request: zahra.karimian@uni-heidelberg.de.
